# SPARK: Supporting Psychiatric Academic Research for Knowledge. a Quality Improvement Project Designed to SPARK Interest in Research in the West Midlands Deanery

**DOI:** 10.1192/bjo.2026.11518

**Published:** 2026-06-30

**Authors:** Chantelle Wiseman, Amitav Narula, Angharad de Cates, Andy Owen, Ravindra Belgamwar

**Affiliations:** 1 University of Birmingham, Birmingham, United Kingdom; 2 Black Country Healthcare NHS Foundation Trust, Wolverhampton, United Kingdom; 3 Coventry and Warwick Partnership NHS Trust, Coventry, United Kingdom; 4 North Staffordshire Combined Healthcare NHS Trust, Stafford, United Kingdom

## Abstract

**Aims:**

To improve higher psychiatry resident doctors’ access to research opportunities in the West Midlands Deanery.

The updated training curricula in 2022 requires higher psychiatry resident doctors to demonstrate involvement with ethically approved research studies. Some residents were struggling to achieve this competency.

**Methods:**

We commenced a QI project in November 2024 to support residents with research opportunities. We have implemented these four approaches.

1. Working Group: we meet as a working group every few months, chaired by the Head of School, to discuss strategy.

2. Research project document: we compiled a list of ethically approved research projects available in the region with contacts for each. This is accessible to resident doctors in the region via the learning platform PGVLE. We are currently on the second version and plan to update this document annually.

3. Linking residents with projects: we educate resident doctors on their research requirements at their teaching sessions. We meet with residents 1:1 at their request and link them to research projects in their area of interest.

4. Co-ordinating research engagement: we ensure members of the working group are involved in relevant projects run throughout the region. These include the annual Research Day in the Black Country NHS Trust, the MRCPsych course, the annual Medical Student Psychiatry Summer School in Birmingham, the monthly Regional Training Days for the higher resident doctors, and the funded Research and Academic Network Meetings in Coventry and Warwickshire NHS Trust.

**Results:**

A preliminary survey of satisfaction with the non-clinical elements of training was completed in October 2024 for the West Midlands General Adult Higher Residents (n=36) and repeated in October 2025 (n=11).

In 2024, 42% said they were somewhat or extremely unconfident in their knowledge about the portfolio requirements for research; in 2025 this was reduced to 18%. In 2024, 69% said they had not been given adequate opportunities to meet research training requirements; in 2025 this was 45%.

**Conclusion:**

We have been working to support resident doctors in the West Midlands to achieve their research competencies during higher training. Satisfaction with the awareness of research requirements and the opportunities to achieve them have increased. We believe these approaches can be used in other regions.

